# High-pressure processing of pork liver reduces the infectivity of the hepatitis E virus

**DOI:** 10.1128/aem.01054-25

**Published:** 2025-12-31

**Authors:** Marie Pellerin, Jean-Luc Martin, Lauranne Harlet, Virginie Doceul, Nicole Pavio, Carole Feurer

**Affiliations:** 1 Anses, INRAE, EnvA, UMR Virologie 52839, Maisons-Alfort, France; 2 Department of fresh and processed, IFIP-Institut du Porc 91540, Paris, France; 3 Department of fresh and processed, IFIP-Institut du Porc, Pacé, France; Anses, Maisons-Alfort Laboratory for Food Safety, Maisons-Alfort, France

**Keywords:** hepatitis E virus, zoonosis, high-pressure processing, pork liver sausage, food safety

## Abstract

Hepatitis E virus (HEV) causes acute and chronic hepatitis in humans. The zoonotic HEV genotype 3 (HEV-3) is present in various animal species, including pigs, wild boars, and other game animals. Foodborne transmission with the consumption of raw or undercooked pork products is the major transmission route of HEV-3. HEV RNA has been detected in various types of food, but particularly in pork liver-based food products. High hydrostatic pressure processing (HPP) can be used for the inactivation of pathogens in food. In the present study, the impact of HPP treatments was evaluated on HEV-3 infectivity in raw pork liver. Different pressure/time combinations (500 MPa for 1 or 5 min, 600 MPa for 1, 5, or 10 min) were applied to raw pork livers, artificially contaminated with HEV-3 (8.3 log_10_ HEV ge/g). Residual HEV infectivity was evaluated using the HepaRG cell culture model in p-24 well plates. The results obtained have shown the absence of residual infectious HEV particles in pork liver after a treatment of 600 MPa, during 1 min in a refrigerated room at +8°C. Then, liver sausages were prepared with pork liver treated at 600 MPa for 1 min. Technological measurements showed that the treatment had a significant impact on brightness, firmness, red hue, and cohesiveness. Nevertheless, these differences have not been perceived after food testing, which highlighted no major difference in taste or color. Thus, inactivation of HEV-3 in raw pork liver by HPP is a possible treatment to limit the risk of HEV exposure through food consumption.

**IMPORTANCE:**

The hepatitis E virus (HEV) is the leading cause of enterically transmitted acute hepatitis worldwide. It can have a zoonotic origin through the consumption of infected meat. Pigs are the main reservoir of zoonotic HEV, and pork livers are frequently contaminated by HEV. In the present study, we investigated the use of high-pressure processing (HPP) to inactivate HEV-3 in pork liver. This study is the first to identify HPP treatment parameters that can be applied to pork liver to reduce HEV infectivity. Additionally, it is the first study to demonstrate the feasibility of processing HPP-treated pork liver into food products, such as dry liver sausage.

## INTRODUCTION

Hepatitis E virus (HEV) is a quasi-enveloped single-stranded RNA virus classified in the *Hepeviridae* family, subfamily *Orthohepeviridae* which belongs to the species *Paslahepevirus balayani* (1). HEV is responsible for enterically acquired hepatitis in humans that is often acute and self-limiting but can evolve to fulminant hepatitis or cause chronic hepatitis, particularly in immunocompromised patients (2). A recent study including several hundred viruses with zoonotic potential has classified HEV among the 10 viruses with the highest risk of spillover from wild fauna (3).

Four major HEV genotypes have been identified in humans. Genotypes 1 and 2 (HEV-1 and -2) strictly infect humans, while genotypes 3 and 4 (HEV-3 and -4) are zoonotic and largely present in pigs and wild boar reservoirs (4, 5). HEV-3 and -4 infections in humans have a foodborne origin. Although other transmission routes exist, foodborne transmission is considered the primary mode of HEV contamination with the consumption of meat and meat products prepared from infected animals (6). Notably, HEV-3 subtypes 3c and 3f are circulating predominantly in Europe in both animal and human populations (7).

In recent years, the incidence rate of HEV-3 infection has increased in industrialized countries (8). Several studies have demonstrated a high prevalence of anti-HEV antibodies in the human populations of various European countries, with studies carried out in France and Germany showing seroprevalences over 20% (9, 10).

Evidence indicates that infection with HEV-3 is common among domestic swine. High prevalences of antibodies have been detected in European swine populations (11, 12).

Studies carried out in pigs at the slaughterhouse have shown that a significant proportion of the animals and their organs, particularly the liver, are contaminated with HEV. For example, in France, 2%–4% of pork livers collected at slaughterhouses were found to be positive for HEV RNA (11, 13).

HEV RNA was detected in various types of food, but particularly in pork liver-based food products (14). The prevalence of contaminated pork products varies from 0.03% to 50% depending on the geographical region and tested meat products (15–20).

In human populations, higher prevalence of HEV IgG has been observed in regions where products made from uncooked pork liver are consumed, for example, in several regions of France and Italy (9, 21).

Because of the high prevalence of HEV RNA in food products containing raw pork liver, efficient strategies to inactivate HEV are necessary. HEV can survive for a long time in food products with only a slight decrease in infectivity (22, 23). Certain traditional preservation methods, such as drying, are not significantly effective for HEV inactivation (24). High concentration of nitrite salt, often used to cure sausage, seems to be tolerated by HEV, as well as pH variations (25).

The easiest method for reducing HEV contamination is heating, and its efficiency has been shown in several studies (26, 27). However, not all types of food products can be heated, and in particular, dried or fresh liver sausages, one of the main sources of HEV exposure (15). Thus, alternative methods for HEV inactivation should be developed for specific food products.

High pressure processing (HPP) is a “non-thermal pasteurization” technique that has already demonstrated hundred years ago its efficiency for inactivating microorganisms, particularly in milk (28). Currently, HPP is used as a gentle alternative to thermal treatment in various food products such as juice, vegetables, and seafood products (29). The inactivation effect is multifactorial, due to pH change, protein denaturation, permeabilization, and/or modification in fluidity of cell membranes (30, 31).

HPP treatment has also been shown to be useful for inactivating several viruses, with minimal influence on the physicochemical and sensory properties of foods (32). The HPP treatment effect on viruses is dependent on the food type and its components. The inactivation behavior can be more or less important according to the protective role of the food matrix. Thus, for feline calicivirus and murine norovirus, a higher reduction is measured in swine liver rather than in ham after the same HPP treatment (400 MPa/10 min) (31). Different HPP inactivation effects have also been shown between chicken meat and eggs for bursal disease virus (33). Even if HPP does not impact covalent bonds, quality properties of food can be affected, depending on the HPP conditions, treatment, and food type treated (34, 35). Thus, testing needs to be done for each specific food matrix.

Recent studies have explored the effect of HPP on HEV inactivation in different matrices. An initial study was carried out in culture medium and liver pâté (36). A 2-log decrease in infectivity of HEV was observed in culture medium at 400 or 600 MPa for 1 or 5 min, and a 0.5-log decrease in liver pâté. A second study was carried out on the inactivation of HEV in phosphate-buffer suspension (PBS) (37). A gradual decrease in infectivity was observed by application of 100 to 600 MPa for 2 min. The virus was nearly completely inactivated at 600 MPa. At last, one study analyzed the effect of HPP on HEV inactivation in human milk (38). Their results demonstrated that HEV was not affected after moderate HPP treatments, and the milk matrix did not fully protect HEV from inactivation at 600 MPa.

Given the varying results obtained depending on the nature of the matrices, it is important to further establish an effective HPP treatment against HEV in raw pork liver.

To this end, we investigated the impact of different pressure/time combinations on HEV infectivity in raw pork livers artificially contaminated with HEV-3. This was achieved using the cell culture HepaRG cells and the adapted genotype 3f strain FR-HuHEVF3f. Then, industrial partners have assessed the technological and sensory properties of liver-dried sausages prepared with PPH-treated pork liver.

## MATERIALS AND METHODS

### Cells and virus

Human HepaRG cells were grown as previously described (39). Cells were seeded into 24‐well plates (5 × 10^4^ cells/well). They were maintained in growth medium for 2 weeks, and medium with 1.2% DMSO was replaced for two extra weeks for cell differentiation into hepatocytes. After 4 weeks, cells were infected overnight with virus inoculum under a maximum volume of 250 μL. The viral suspension was then removed, and cells were washed three times in PBS before adding 0.5 mL of growth medium. Fresh medium was renewed every 2–3 days. Supernatants from infected cells were collected once a week for HEV RNA detection by RT‐qPCR.

The HepaRG cell model can be used to detect infectious HEV from naturally contaminated liver samples (40).

The HEV genotype 3f strain FR-HuHEVF3f (GenBank JN906974), originally derived from a French patient suffering from acute autochthonous hepatitis E, was used for all spikings and infections. Six consecutive passages of the virus were previously carried out in HepaRG (39).

### Virus production and sample inoculation

To obtain a high viral load and sufficient viral stock for all infections, HepaRG cells were infected (passage 6) in T75 flasks with 3.5 mL (8.7 log_10_ HEV ge/mL) overnight. Then, the inoculum was removed, cells were washed three times with PBS, and fresh medium was added. Supernatants were collected three times per week for 50 days, from day 65 to day 115, when the medium was renewed. All virus supernatants were pooled (around 960 mL with 8.5 log_10_HEV genome equivalent in RNA copy numbers (HEV ge/mL). To keep a homogeneous viral population of quasi-enveloped HEV particles, intracellular particles were not collected.

In order to concentrate the virus, the pooled supernatants were centrifuged on a Vivaspin20® centrifugal concentrator (100 kDa). A viral stock suspension of 70 mL was obtained, with a concentration of 9.4 log_10_ HEV ge/mL.

Liver from a specific pathogen-free pig, not infected by HEV, was cut into 1 cm^3^ pieces and divided into 36 plastic bags, with approximately 15 pieces of liver per bag (20 g). In 30 bags, liver samples were artificially contaminated by intra-tissular injection with needles (Microlance3 Becton Dickinson, 0.3 × 13 mm) of viral suspension (2 mL), corresponding to 100–150 µL inoculum per piece, equivalent to 8.3 log_10_ HEV ge/g of liver. The other six liver samples were injected with culture medium as a negative control.

Each sample was vacuum-packed and placed in a sealed plastic bag. It was then placed in a third plastic bag containing a solution of peracetic acid to comply with the hygiene and biosafety conditions imposed by the infectious nature of HEV.

### High-pressure treatment

#### Treatment of artificially contaminated samples

For each HPP condition tested, samples included five HEV artificially contaminated livers, one control liver not contaminated with HEV, and three viral suspensions corresponding to the viral stock diluted 1:10 in 2 mL PBS.

The HPP treatment was carried out on a 100-L horizontal high-pressure equipment (Avure AV-10) located on an industrial platform, in a refrigerated room at +8°C. The pressure-transmitting fluid used was water, whose temperature ranged from 7.1°C to 9.0°C before treatment. The compression rate was 4.26 MPa·s^−1^, and decompression was nearly instantaneous. During HPP treatment, the cylinder temperature increased because of the adiabatic heating, without exceeding 26°C.

The HPP scales tested were chosen based on the literature and, in particular, in relation to scales enabling hepatitis A virus inactivation (41, 42). Samples were exposed to different pressure levels as follows: 500 MPa for 1 and 5 min, 600 MPa for 1, 5, and 10 min. The scale of 600 MPa for 10 min corresponded to a positive control of inactivation. After treatment, the pressurized samples were placed at +4°C in a cold room before direct shipping at +4°C for infectivity analysis. Shipment time lasted no longer than 1 day (24 h).

#### Treatment of pork liver for liver sausage processing

The HPP treatment, allowing total inactivation of the HEV virus, was applied to 30 kg of fresh vacuum-packed pork livers. High-pressure-treated livers were used to make liver sausages on an industrial scale by two partner companies, A and B. The formulation of the liver sausages slightly differed between companies and was as follows: company A: pork meat, pork liver (30%), salt, lactose, dextrose, spices, flavor enhancer, stabilizer, preservatives, flavors, smoke flavors, starter cultures, and natural pork casing; company B: pork meat and fat, pork liver (30%), salt, lactose, dextrose, milk powder, spices, preservatives, starter cultures, and natural pork casing. The sausages were dried for 15 days. On the finished product, the fat content was 29.4 g/100 g for company A and 35 g/100 g for company B. The organoleptic quality of the products was assessed by both companies by an internal expert panel composed of the General Manager, the Quality Manager, the Production Manager, and the Production Technician. All of them are routinely involved in evaluating newly developed in-house liver sausage formulations, as this activity forms an integral part of their professional duties. In this specific context, an Institutional Review Board is not required. The evaluation was performed according to the following criteria: appearance, color, taste, and texture. At the same time, technological measurements (see below) were carried out on both productions of liver sausage with the casing removed. The results were compared with a control production made under the same conditions with livers not treated by HPP, in each case.

### Technological measurements

#### Color measurements

Color measurements were performed using a KONICA MINOLTA CM600-d spectrocolorimeter, for an 8 mm diameter measuring area, including specular mode, in CIELAB color space. The measured values were the brightness (L*) and red hue angle, in degrees (H*). Thirty independent measurements per partner company were performed on five independent liver sausages at a rate of 6 measurements per sausage.

#### Texture measurements

Texture measurements were carried out using a TA-XTPlus texturometer equipped with a round-section compression tool. They were carried out on a sample of 15 mm in diameter and 10 mm in height, after removal of the casing. Six measurements were carried out on five sausages treated (*n* = 30) or not (*n* = 30) per company with the TPA method: double compression at constant speed on 7.5 cm (75%) in the center of the slice.

The values considered were firmness (N) and cohesiveness, the best representative indices of coarse-grained products.

#### Data processing

Data were processed by variance analysis using R 4.3.2 software. The variances' homogeneity was confirmed using the Bartlett test with a threshold of 0.5%. The normality of the data dispersion was confirmed using the Shapiro-Wilk test with a threshold of 0.5%. The effects of company and treatment factors were determined with a threshold of 5%. Significantly different groups were determined using the Tukey-C test. Measurement dispersion was highlighted using ggplot2 graphs.

### HEV infectivity assay

After HPP treatment and shipping, all samples were stored at −80°C until analyses. Three independent experimental infections were carried out in triplicate.

Livers were homogenized twice with 40 mL PBS, using a blender. Two successive centrifugations were performed to eliminate the food matrix debris as much as possible. Supernatants were diluted in cell medium (1/20) and passed through a 0.45 µM and then a 0.22 µM pore size filter.

After the final step of inoculum preparation (homogenization, centrifugation, dilution, and filtration), the titers of the suspensions were 6.3 log_10_₀ HEV ge/mL instead of the expected 6.7 log_10_ HEV ge/mL. Thus, half of the viral load was recovered.

For each liver suspension, three independent infections were carried out on DMSO-differentiated HepaRG cells. Infections were maintained for up to 42 days, and cell culture medium was changed three times a week. Supernatant samples were collected once a week to quantify HEV RNA production.

The limit of infectivity of HEV culture *in vitro* (5.3 log_10_HEV ge/mL) was defined by carrying out serial dilutions of viral suspensions and optimizing the culture conditions of HepaRG cells (See Fig. S1 at https://doi.org/10.57745/MNRNYA). For each series of infections, a positive culture control was performed with untreated virus in suspension corresponding to 5.3 log_10_HEV ge/mL. Statistical analysis was performed using GraphPad Prism v.9.0 (GraphPad Software) and the Kruskal-Wallis test method.

### HEV RNA isolation and quantification

RNA extraction from liver homogenate, PBS suspension, or cell culture supernatant was performed using a magnetic bead‐based separation technology and the KingFisher Duo (Thermo Fisher Scientific, Courtaboeuf, France). Total RNAs from 200 µL supernatant were extracted using the MagMAX core nucleic acid purification kit (Thermo Fisher Scientific, Courtaboeuf, France), according to the manufacturer’s instructions and the KingFisher instrument guide (40). Elution was performed in 90 μL of RNAse-free water.

HEV RNA detection and quantification were performed using a real‐time quantitative RT‐PCR as previously adapted from a method described by reference 43 in reference 26.

A standard quantification curve was obtained using *in vitro* transcribed RNAs from the plasmid pCDNA 3.1 ORF2‐3 HEV. The limit of detection of the applied system is 3.3 log_10_ HEV ge/mL and 4.8 log_10_ HEV ge/g, corresponding to five copies of HEV RNA in 2 μL of total RNA extract.

## RESULTS

The impact of different pressure/time combinations on HEV-3 infectivity was investigated in pork livers artificially contaminated with FR-HuHEVF3f and in viral PBS suspensions, treated at 500 MPa for 1 and 5 min, and at 600 MPa for 1, 5, and 10 min.

### Effect of HPP treatment on the molecular quantification of HEV RNA by RT-qPCR

First, the impact of high-pressure treatments on the molecular detection of HEV was evaluated by quantifying HEV RNA genome equivalent in copy numbers (HEV ge) by RT-qPCR in HEV-contaminated samples (liver homogenate or PBS suspension) subjected or not to various HPP treatments. Initial HEV RNA titers in pork liver and PBS solutions were, respectively, 6.3 log_10_ and 8.3 log_10_ HEV ge/mL.

The results showed that the various high-pressure treatments tested did not have any impact on the molecular quantification of HEV ge/mL by RT-qPCR. HEV ge/mL was equivalent in treated and untreated samples, both in livers and in viral PBS suspensions (Table 1).

**TABLE 1:** Quantification by RT-qPCR of HEV RNA copy number (in log_10_) per milliliter of liver homogenate or PBS suspension (log_10_ HEV ge/mL), after HPP treatment or not, and before infection of HepaRG cells (mean of three independent samples ± standard deviation)

	log_10_ [HEV ge/mL] (mean ± SD)	
	Liver homogenate	PBS suspension
Untreated	6.3 ± 0.1	8.2 ± 0.1
500 MPa/1 min	6.5 ± 0.2	8.3 ± 0.2
500 MPa/5 min	6.6 ± 0.2	8.2 ± 0.2
600 MPa/1 min	6.3 ± 0.2	8.2 ± 0.1
600 MPa/5 min	6.4 ± 0.3	8.3 ± 0.2
600 MPa/10 min	6.5 ± 0.2	8.2 ± 0.2

### Evaluation of HEV residual infectivity in pork liver matrix subjected to HPP using HepaRG cell cultures

**Fig 1:**
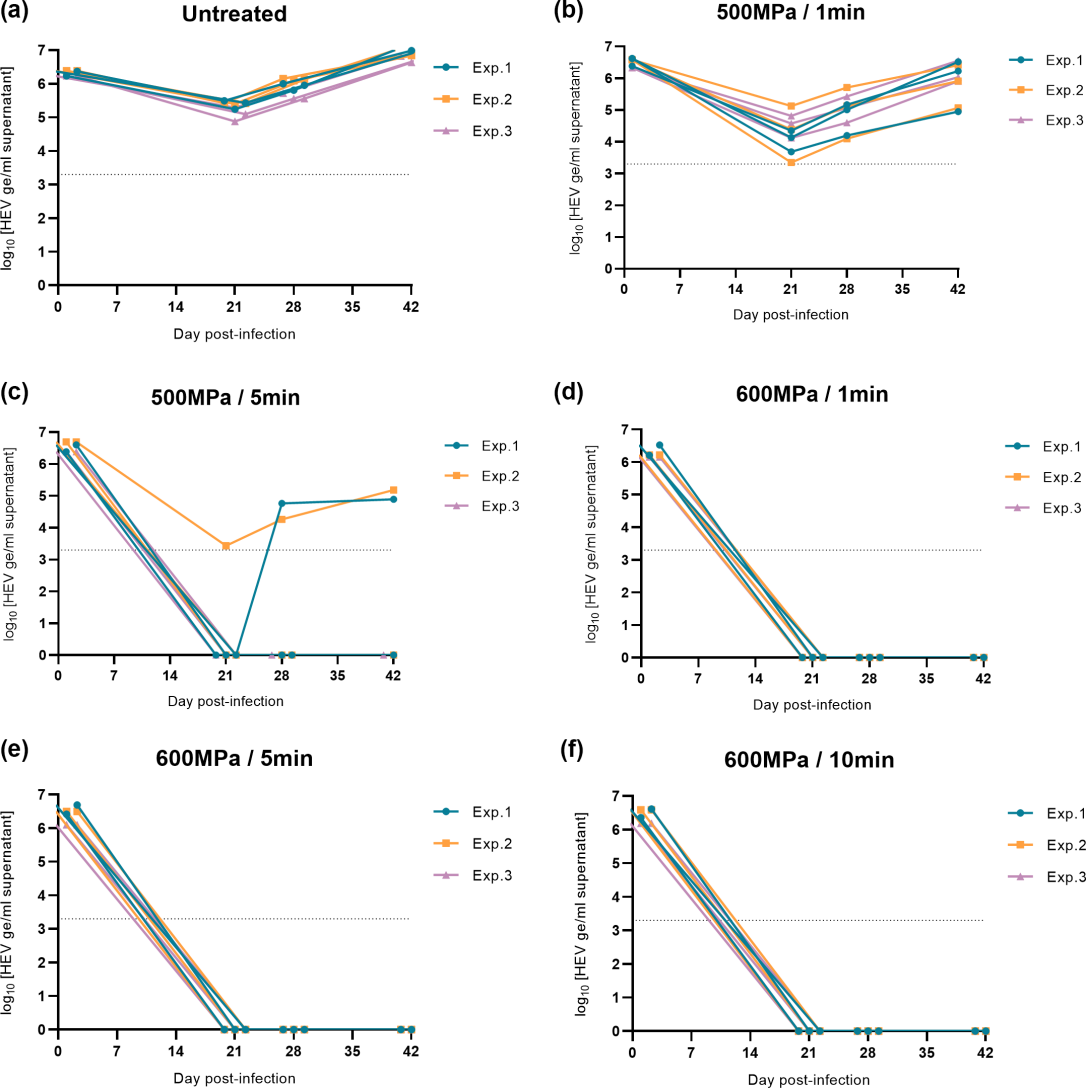
HEV replication kinetics in HepaRG cells during 42 days post-infection. HEV RNA quantity was measured in culture supernatants after infection with a homogenate of infected pork liver samples treated or not with different HPP parameters. (**a**) Untreated, (**b**) 500 MPa/1 min, (**c**) 500 MPa/5 min, (**d**) 600 MPa/1 min, (**e**) 600 MPa/5 min, and (**f**) 600 MPa/10 min (log_10_ HEV ge/mL). For each condition, three independent experiments were performed in triplicate (Exp. 1 blue dot, Exp. 2 orange square, and Exp. 3 lilac triangle). The limit of detection of the RT-qPCR assay is represented by the dotted line.

In order to determine if high-pressure treatments have an impact on HEV infectivity, homogenates of liver samples subjected to HPP or not were cultured in HepaRG cells. This model allows detecting the presence of infectious HEV in samples by monitoring viral RNA production in the supernatant of infected cell cultures for 6 weeks (42 days), using RT-qPCR (40). Three independent experiments were carried out (independent liver bags treated), with three cell culture wells infected per condition for each experiment (Fig. 1).

In order to avoid the measurement of residual inoculum, cell supernatants were collected and analyzed during 6 weeks post-infection (42 days). HEV replication in cell culture was considered positive when an increase in RNA HEV level was observed during this period of time. Without treatment, the quantity of HEV RNA increased in cell supernatant between 21 and 42 days post-infection (dpi), demonstrating HEV replication (Fig. 1a). The HEV RNA measures (ge/mL) in the supernatants of the three independent experiments show variability in the levels of HEV produced of unknown origin, confirming the necessity of performing several replicates. HEV RNA was detected after 42 dpi in the supernatant of 1 to 3 cultures of HepaRG cells infected with livers treated at 500 MPa for 1 or 5 min, showing the presence of infectious viruses in these samples. No HEV RNA was detected after 42 dpi in the supernatant of HepaRG cells infected with livers treated at 600 MPa for 1, 5, and 10 min, suggesting the decrease of infectious HEV-3, under the limit of detection in these samples (Table 2). Considering that the cell culture system used allows the detection of 5.3 log_10_ HEV ge/mL (See Fig. S1 at https://doi.org/10.57745/MNRNYA), at least a one-log reduction was reached with 600 MPa HPP from 1 min.

**TABLE 2:** Number of HepaRG cultures with detectable HEV RNA in the supernatant at 42 days post-inoculation (assessed by RT-qPCR) relative to the total number of inoculated cultures derived from infected livers, either untreated or subjected to HPP (500 MPa for 1 or 5 min, or 600 MPa for 1, 5, or 10 min)^*a*^

	Number of positive cultures for HEV replication			
	Exp. 1	Exp. 2	Exp. 3	TOTAL
Untreated	3/3	3/3	3/3	**9/9**
500 MPa/1 min	3/3	3/3	3/3	**9/9**
500 MPa/5 min	1/3	1/3	0/3	**2/9**
600 MPa/1 min	0/3	0/3	0/3	**0/9**
600 MPa/5 min	0/3	0/3	0/3	**0/9**
600 MPa/10 min	0/3	0/3	0/3	**0/9**

^*a*^ Grey cells indicate positive results.

**Fig 2:**
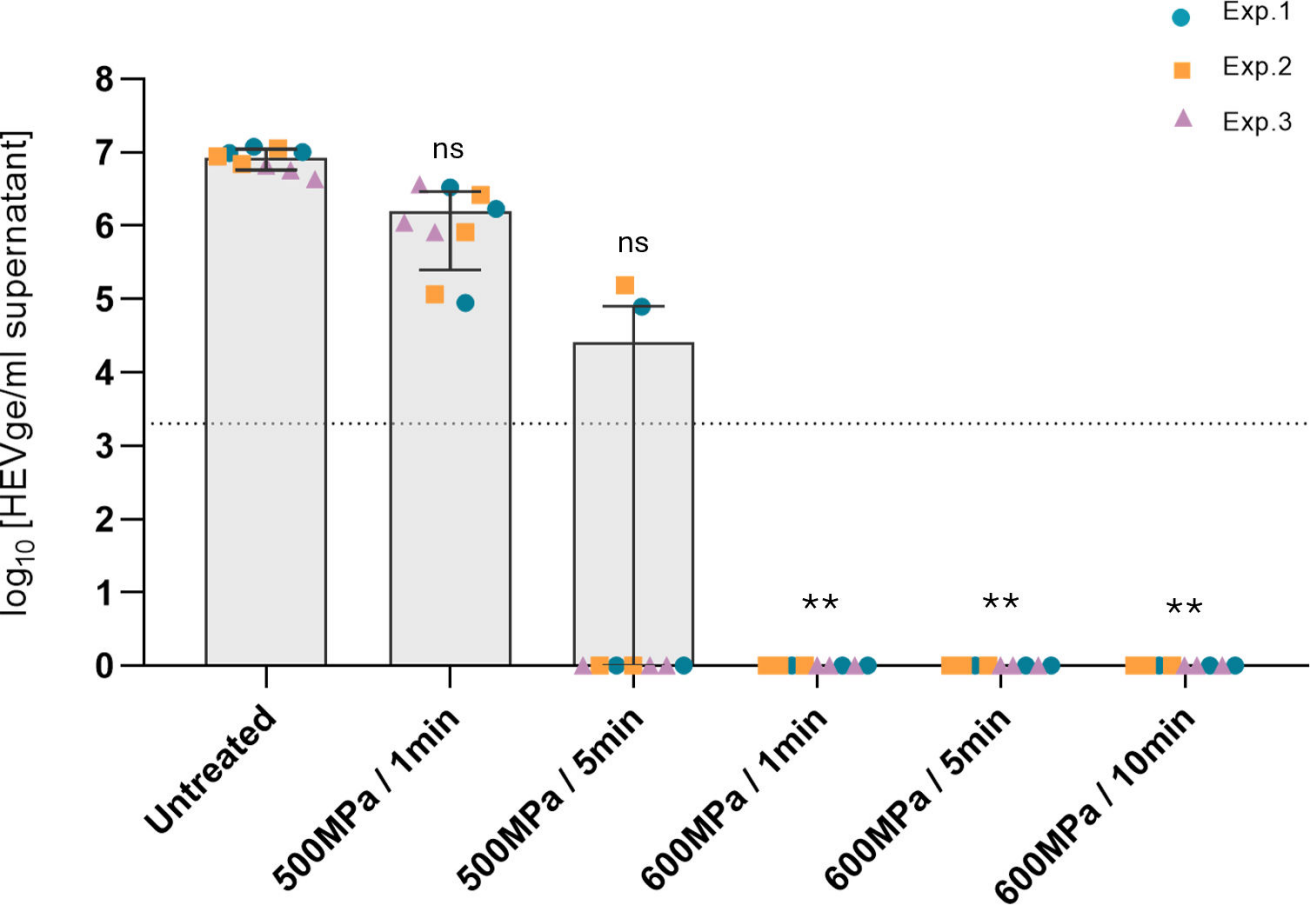
Quantity of HEV RNA (in log_10_ HEV ge/mL) in the supernatants of HepaRG cells at 42 days after infection with homogenate of infected pork liver samples treated 500 MPa during 1 or 5 min, or 600 MPa during 1, 5, or 10 min, or untreated (atmospheric pressure). Mean (±SD) of nine samples (three experiments performed in triplicate: Exp. 1 blue dot, Exp. 2 orange square, and Exp. 3 lilac triangle). Limit of detection of the RT-qPCR assay is represented by the dotted line. Statistical analysis, with the Kruskal-Wallis test, was applied in comparison to the untreated samples; non-significant (ns) and significant differences ** (*P* < 0.005) are indicated.

In the HepaRG cell culture model of HEV, it is possible to link the quantity of virus produced in the supernatants after 42 days of culture, with the quantity of infectious virus initially present (See Fig. S2 at https://doi.org/10.57745/MNRNYA). At 500 MPa, the quantity of HEV RNA increased in cell supernatant between day 21 and day 42, but the quantity of HEV RNA measured in culture supernatants at 42 days was lower in samples treated at 500 MPa than in untreated samples (by up to 2 log) (Fig. 2). This decrease was more important when the treatment time increased from 1 min with 1 log reduction and 9 HEV positive cultures out of 9 to 5 min with at least 2 log reduction and 2 HEV positive cultures out of 9. This suggests a partial effect of the 500 MPa treatment on the inactivation of HEV in pork liver.

### Evaluation of HEV residual infectivity in PBS suspension subjected to HPP using HepaRG cell cultures

In order to evaluate a possible effect of the liver matrix on HEV inactivation, the same HPP pressure/time combinations were applied to viral suspensions diluted in PBS buffer.

As above, HepaRG cells were infected with viral suspension diluted in PBS, treated or not with the same HPP conditions. As for liver sample infections, HEV RNA was monitored in cell supernatant for 42 days. Infections were carried out with the initial viral suspension with 8.3 log_10_ HEV ge/mL and two dilutions (1/10 and 1/100) of this suspension, corresponding to 7.3 log_10_ and 6.3 log_10_ HEV ge/mL. The latest dilution allowed comparison of the results obtained with liver samples artificially contaminated with 6.3 log_10_ HEV ge/mL. After infection with viral suspensions with 8.3 log_10_ HEV ge/mL of HEV-3, treated at 500 MPa (1 and 5 min), HEV RNA replication was detected in all cell cultures (*n* = 6). However, no HEV replication was observed in cultures infected from suspension treated at 600 MPa, whatever the treatment time (1, 5, or 10 min) (*n* = 6) (Table 3; See Fig. S3 at https://doi.org/10.57745/MNRNYA). A suggestion that a log reduction of >3 was reached in PBS suspension.

**TABLE 3:** Number of HepaRG cultures with positive HEV RNA replication (detected by RT-qPCR) at 42 days post-inoculation relative to the total number of inoculated cultures with viral suspensions in PBS buffer (8.3 log_10_ HEV ge/mL) subjected to HPP (500 MPa during 1 or 5 min or 600 MPa during 1, 5, or 10 min), or not, 42 days post-inoculation^*a*^

	Number of positive cultures for HEV replication			
	Exp. 1	Exp. 2	Exp. 3	TOTAL
Untreated	2/2	2/2	2/2	**6/6**
500 MPa/1 min	2/2	2/2	2/2	**6/6**
500 MPa/5 min	2/2	2/2	2/2	**6/6**
600 MPa/1 min	0/2	0/2	0/2	**0/0**
600 MPa/5 min	0/2	0/2	0/2	**0/0**
600 MPa/10 min	0/2	0/2	0/2	**0/0**

^*a*^ Green cells indicate positive results.

Data with the 7.3 log_10_ HEV ge/mL viral suspensions are shown in Fig. S3 at https://doi.org/10.57745/MNRNYA. The amount of virus produced after infection with suspensions treated at 500 MPa for 1 min was lower, with 2 log reduction and 6 HEV positive cultures out of 6, than that of untreated suspensions, indicating a lower amount of residual virus after treatment. The inactivation effect was enhanced when the treatment was increased from 1 to 5 min, with no detection with the 500 MPa/5 min treatment (*n* = 6), suggesting a >3 log reduction.

**Fig 3:**
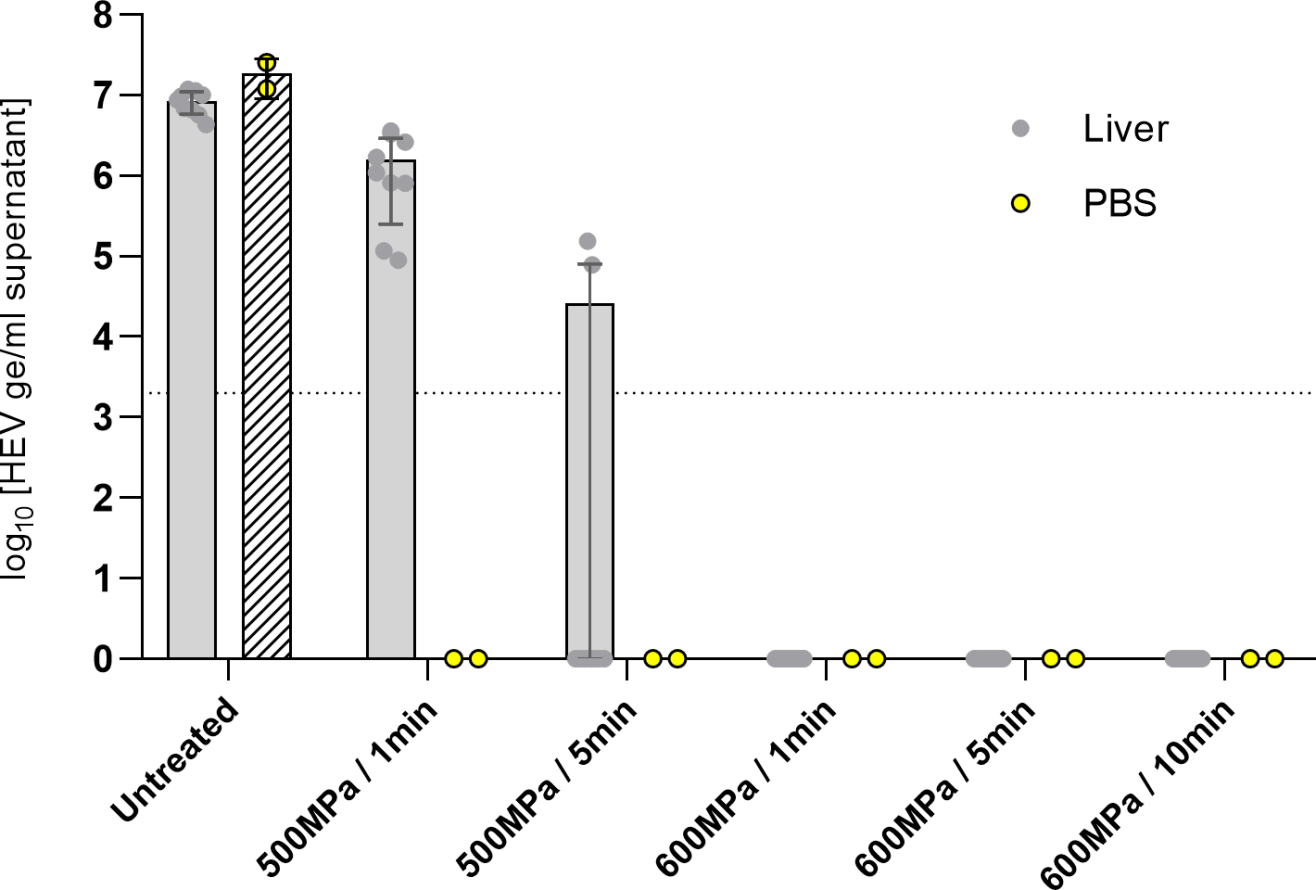
Quantification of HEV RNA (log_10_ HEV ge/mL) in HepaRG cell supernatants 42 days after infection with infected liver (gray dots) (*n* = 9) or viral PBS suspension (yellow dots) (*n* = 2) with 6.3 log_10_ HEV ge/mL, according to the HPP (500 MPa during 1 or 5 min or 600 MPa during 1, 5, or 10 min). The bar graph corresponds to the mean (±SD) and circles to different samples. The limit of detection of the RT-qPCR assay is represented by the dotted line.

For infections, with 6.3 log_10_ HEV ge/mL in PBS, no viral replication could be detected in any culture infected with HPP-treated suspensions in all conditions (Fig. 3). In the liver infected with 6.3 log_10_ HEV ge/mL, infectious virus could be detected after treatment at 500 MPa for 1 and 5 min. Thus, for an equivalent initial viral load, more infectious HEV virus remained when the treatments were applied to the liver matrix than in the PBS suspension (Fig. 3).

### Technological measurements

The impact of the HPP treatment inactivating HEV at 600 MPa for 1 min was assessed on the technological and sensory properties of dried liver sausages produced with HPP-treated pork liver by industrial partners. Technological measurements (dispersion of color and texture measurements) were carried out on pilot productions, and the organoleptic quality of the products was assessed by both companies.

### Dispersion of color measurements

**Fig 4:**
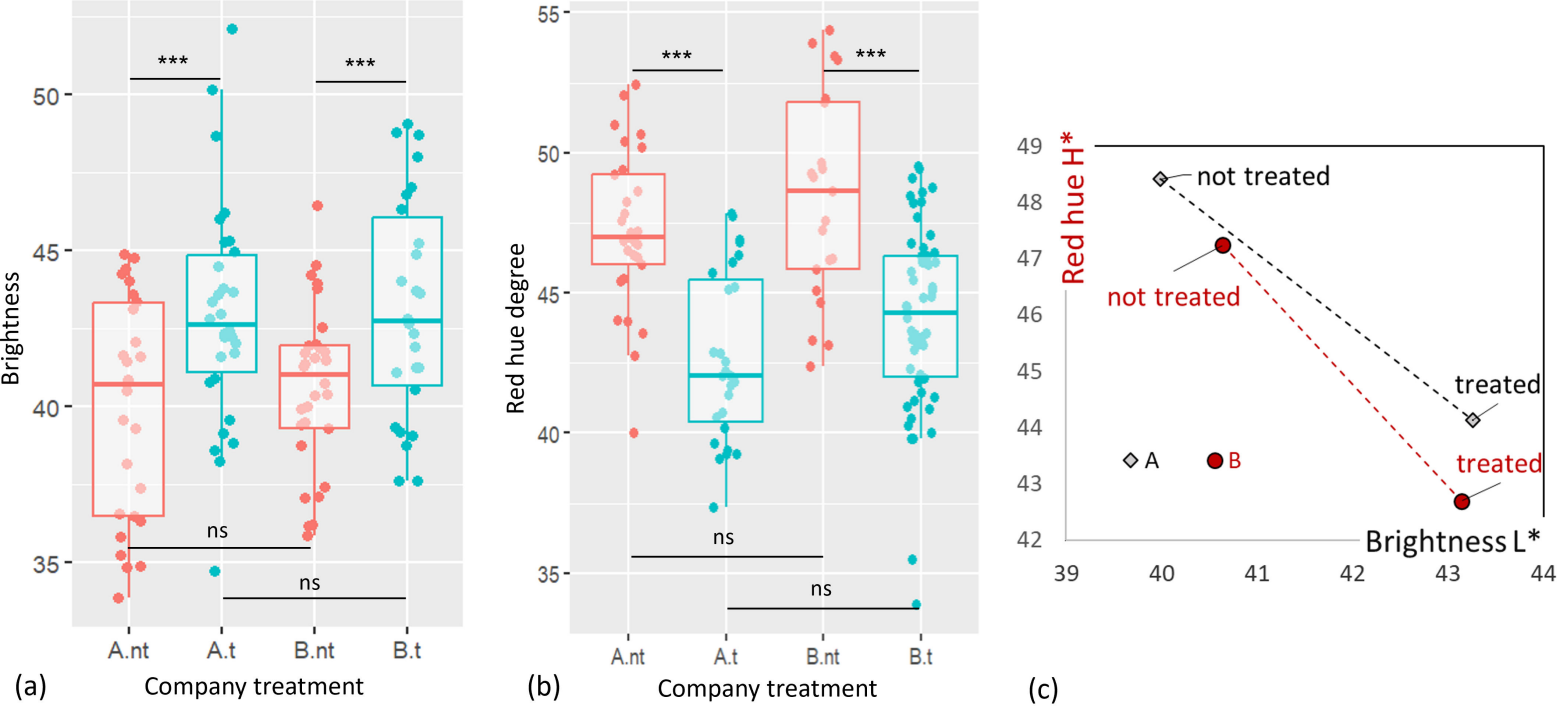
Dispersion of color measurements, brightness, and red hue (degree) indexes, for treated (t, in blue) and not treated (nt, in red) liver sausages of each company A and B. (**a**) Box plot of the brightness measures, (**b**) box plot of the red hue degree measures, and (**c**) positioning of products according to colorimetric indices, companies, and treatment levels (red hue in red; brightness in black). Box plots (**a**) and (**b**) were created with R using ggplot2 module. Graph (**c**) was created using EXCEL software. *** Means that *P* < 0.001 ; ns : non-significant.

For each company, brightness and red hue measurements of not treated versus treated sausages are presented in Fig. 4. Despite the heterogeneity of the sausages composed of large grains of meat and fat, the dispersion of the 30 color measurements is not significant regardless of the sausage product (treated or not) or the company. Indeed, the coefficient of variation ranged from 7% to 9% for brightness and from 6% to 8% for red hue.

The analysis of variance showed that there was no significant effect of the factor “company” on the brightness and red hue (*P* > 1). The factor ‘’company’’ includes all manufacturing parameters such as raw materials, ingredients, additives, and the technological process. However, the factor “treatment” had a significant effect on both measures (*P* ≤ 0.001). For both companies, treated sausages were brighter and less red than untreated ones.

Figure 4c clearly shows that the colorimetric indices of untreated sausages from both companies were very close, and that they were essentially differentiated by the treatment applied.

The limit deviation of the human eye’s perception of brightness and red hue is generally 5 units. In this case, the difference in brightness between the two treatment levels should be considered as not perceptible to the naked eye. In our case, the difference in red hue is just at the limit of visual perception. The treatment neither greatly improved the brightness of the sausages nor greatly deteriorated their red hue.

### Dispersion of texture measurements

**Fig 5:**
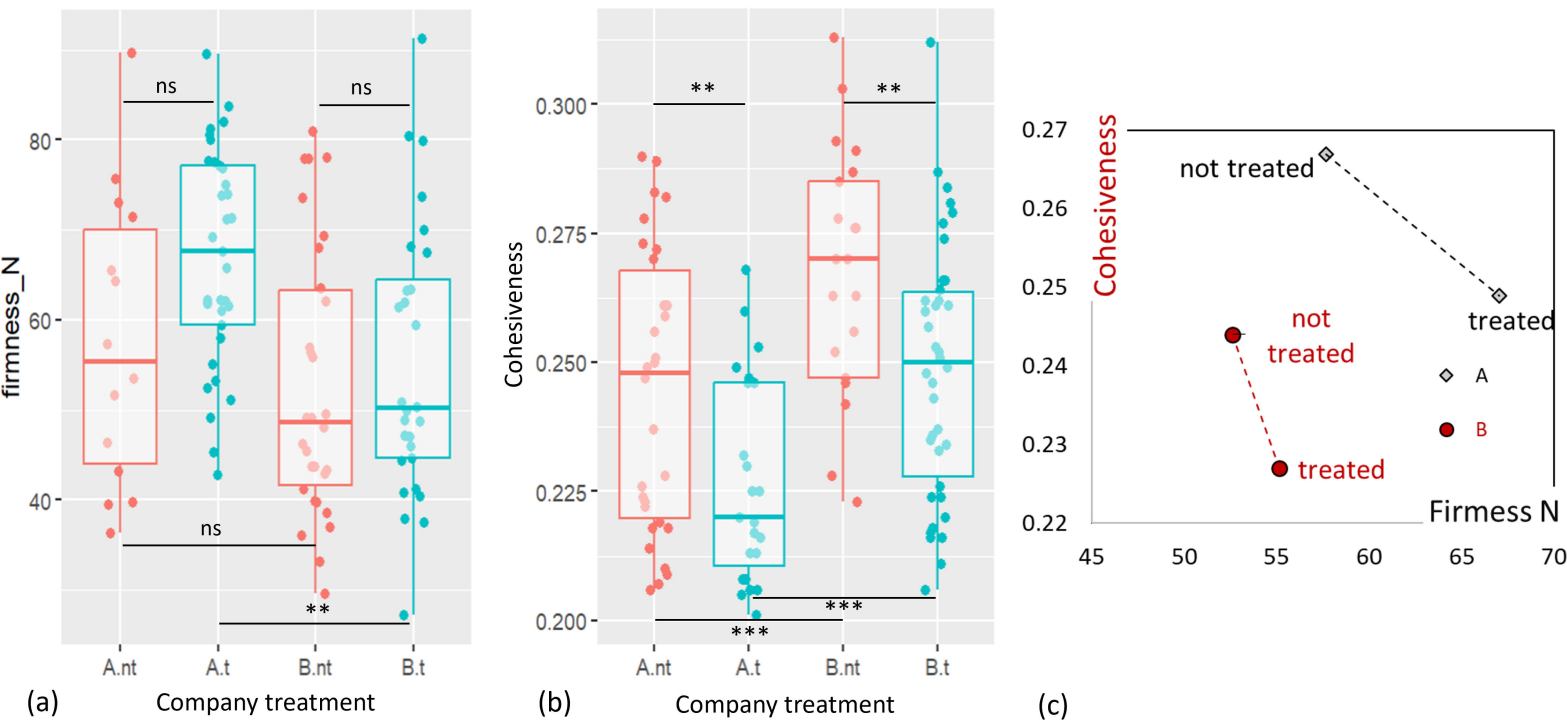
Dispersion of texture measurements, firmness (Newtons), and cohesiveness, for treated (t, in blue) and not treated (nt, in red) liver sausages of each company A and B. (**a**) Box plots of firmness measures N, (**b**) box plots of cohesiveness measures, and (**c**) positioning of products according to texture, companies, and treatment levels (cohesiveness in red; firmness in black). Box plots (**a**) and (**b**) were created using the ggplot2 module. Graph (**c**) was created using EXCEL software. *** Means that *P* < 0.001; ** means that *P* < 0.01; ns : non-significant.

For each company, firmness and cohesiveness measurements of not treated versus treated sausages are presented in Fig. 5. The dispersion of the 30 measurements is greater for firmness (coefficient of variation ranging from 18% to 28%) than for cohesiveness (coefficient of variation ranging from 9% to 11%). Despite the greater dispersion of values for firmness, the Bartlett test confirmed the homogeneity of variances for both firmness and cohesiveness.

There was no significant difference in firmness between both companies regarding untreated sausages, whereas treated sausages from company A were significantly firmer than treated sausages from company B. The characteristics of fresh sausages had a significant effect on the difference in firmness of treated sausages.

For both companies, a decrease in cohesiveness was observed after treatment. Moreover, untreated sausages from company B were significantly more cohesive than untreated sausages from company A. The same difference was observed on treated sausages, highlighting that the treatment alone had a significant effect on the difference in cohesiveness of treated sausages from companies A and B.

Figure 5c shows that for texture indices, differences are linked not only to HPP treatment, but also to the product formulation of the two companies.

As far as texture measurements are concerned, we have no reference for the threshold of human perception of firmness and cohesion.

In Table 4, the global effects of the high-pressure treatment on the four parameters of dried liver sausages of both companies combined are presented. The global effects for the four parameters for each company are presented in Table S1 at https://doi.org/10.57745/MNRNYA.

**TABLE 4:** Overall effects of high-pressure treatment (600 MPa during 1 min) on the characteristics of liver sausages^*a*^^,^^*b*^

	No treatment	Treatment	*P* <
Brightness L*	40.4 ± 3.6 b	43.3 ± 4.0 a	0.001
Red hue H* (°)	48.3 ± 4.4 a	43.2 ± 4.5 b	0.001
Firmness (N)	53.7 ± 17.1 b	62.7 ± 15.8 a	0.01
Cohesiveness	0.25 ± 0.03 a	0.24 ± 0.03 b	0.01

^*a*^ Mean ± SD of data is presented.

^*b*^ Letters (a–b) denote significant differences (*P *< 0.05) between different conditions.

Overall, the high-pressure treatment had a significant effect, which was identical for both product references, on all the parameters studied. The treated sausages were brighter, firmer, less red, and less cohesive than untreated ones.

### Organoleptic evaluation by companies

To assess the impact of HPP treatment, each company called on an in-house panel of four tasters accustomed to consuming liver sausages. The treatment had a visual impact on the liver color, which showed a dull brown color compared to the shiny brown/red control.

Both companies indicated that the mixture before stuffing was paler, but once stuffed and during drying, the sausages regained a color comparable to that of control liver sausages. No major difference in taste in terms of aroma or acidity was observed compared to the control. The slice color was acceptable. Firmness and cohesion were not found to be different compared to the control product.

## DISCUSSION

HPP is used to inactivate microorganisms, including viruses, with minimal influence on the physicochemical and organoleptic properties of food products (29). The inactivation efficiency of HPP on viruses differs according to the viral species, the parameters applied (pressure, time, temperature...), and the composition of the food matrix (32).

As the risk of human exposure to HEV is mainly associated with the consumption of products containing infected pork liver (18–20), the efficacy of HPP treatments was investigated in raw pork liver. For this purpose, pork liver, artificially contaminated with HEV-3, subtype 3f, was submitted to different HPP treatments. The presence of residual HEV particles was evaluated using the HepaRG cell culture system for HEV. The results have shown, first, that the quantification of HEV genome, in the contaminated liver matrix after HPP treatments, was not affected. It confirms that RNA quantification does not reflect the presence of infectious particles. Second, a partial reduction of HEV replication was detected in HepaRG cells with HPP treatments of 500 MPa for 1 min (1 log) or 5 min (2 log). An absence of HEV replication was observed in cell cultures inoculated with pork liver treated at 600 MPa for 1 min.

In comparison to other enteric viruses and surrogate models, HEV showed a high stability to HPP treatments. Indeed, Hepatitis A virus (HAV), murine norovirus 1, or feline calicivirus (FCV) is inactivated, or partially inactivated with treatment from 400 to 500 MPa in various matrices (31, 41, 42).

Then, the effect of the matrix on HEV inactivation was assessed by comparing HEV inactivation in PBS suspensions. In this case, no HEV replication was detected regardless of the HPP treatment applied. Thus, it suggests that the raw pork liver matrix protects HEV from inactivation. HPP induces protein denaturation, membrane permeabilization, and/or alterations in membrane fluidity. Within the hepatocyte/liver microenvironment, HEV particles may interact with cellular factors or the extracellular matrix, potentially attenuating the efficacy of HPP treatment. These results are consistent with other studies, as the matrix nature has been identified as a critical parameter for the design of HPP inactivation strategies (30). For example, FCV and bovine enterovirus, a surrogate for HAV, showed a higher resistance to HPP treatments in mussels and oysters, as compared to seawater and culture medium (44).

Few other studies have investigated the effect of HPP treatments on HEV infectivity in various matrices. In all of them, a high stability of HEV was shown. For example, in liver pâté matrix, a 0.5-log decrease was obtained after a treatment at 400 or 600 MPa for 5 min (36). Another study has analyzed HEV sensitivity to HPP in PBS suspension (37). The authors showed a nearly complete inactivation of HEV at 600 MPa for 2 min. A study on HPP inactivation of HEV in human milk has shown that HEV was inactivated at 600 MPa for 5 min (38).

Overall, HEV inactivation requires high-intensity HPP treatments of 600 MPa for 1 to 5 min. The reduction of infectious HEV measured may vary, depending on the nature of the food matrix (liver pâté, milk, pork liver), the HPP device, the sensitivity of the cell culture system, the method used to detect HEV, or the viral strain used (HEV subtype). Indeed, different strains of HEV may have different inactivation sensitivities to HPP treatments, as it has been shown for six different strains of human rotavirus (45).

It is known that HPP can affect the quality properties of foods (32). Hence, the influence of the HPP treatment on the technological properties of dried liver sausages was analyzed with several parameters. For this purpose, dried liver sausages were prepared with pork liver treated at 600 MPa for 1 min by two independent companies. Technological measurements showed that the treatment had a significant impact on brightness, firmness, red hue, and cohesiveness. Nevertheless, these differences have not been perceived by the companies’ panels, which highlighted no major difference in taste or color compared to the control liver sausage after drying. Moreover, the impact of the treatment on the product’s texture was more likely due to the company’s manufacturing process rather than the HPP treatment itself.

Even if the pilot test has been validated by both companies, the feasibility needs to be confirmed on an industrial scale. Moreover, before HPP treatment of liver becomes part of the production process of dry liver sausages, several bottlenecks must be overcome. For instance, raw liver has to be vacuum-packed before treatment. Ideally, this should be done on an automatic cutting line at the slaughterhouse, which requires a major reorganization of the process. Secondly, transport time between slaughterhouses and companies must be considered as raw liver is a product with a short shelf life that must be quickly handled by food processors. Finally, the additional cost of vacuum packing, HPP treatment, and transport needs to be assessed. Excessive overcost will not be viable for companies.

Our study has certain limitations. First, raw pork liver has been artificially contaminated, and the use of naturally infected pork liver would have been more relevant, although more difficult to collect at the slaughterhouse with an exploitable/sufficient viral load. Secondly, the detection limit of our cell culture model may not allow the detection of small residual amounts of non-inactivated virus. However, it would be important to know the minimal infectious dose for humans following food consumption. Finally, the technological properties of dried liver sausages made with HPP-treated raw pork liver have been analyzed only by two companies. This represents a small number of manufacturers that need to be confirmed on a larger scale.

### Conclusions

In conclusion, this study is the first to establish HPP parameters (600 MPa for 1 min) effective in reducing the presence of infectious HEV in raw pork liver. Moreover, it is the first to demonstrate the feasibility of incorporating HPP-treated pork liver into food products, thereby supporting novel food safety applications.

## EDITOR'S NOTE

**Ed. Note**: A potential conflict of interest was identified after acceptance of this paper, and the editor in chief performed an additional final review of the paper.
